# Preclinical Investigation of PLGA Nanocapsules and Nanostructured Lipid Carriers for Organoselenium Delivery: Comparative In Vitro Toxicological Profile and Anticancer Insights

**DOI:** 10.3390/pharmaceutics18010057

**Published:** 2025-12-31

**Authors:** Bianca Costa Maia-do-Amaral, Taís Baldissera Pieta, Luisa Fantoni Zanon, Gabriele Cogo Carneosso, Laísa Pes Nascimento, Nayra Salazar Rocha, Bruna Fracari do Nascimento, Letícia Bueno Macedo, Tielle Moraes de Almeida, Oscar Endrigo Dorneles Rodrigues, Scheila Rezende Schaffazick, Clarice Madalena Bueno Rolim, Daniele Rubert Nogueira-Librelotto

**Affiliations:** 1 Laboratório de Testes e Ensaios Farmacêuticos In Vitro, Departamento de Farmácia Industrial, Universidade Federal de Santa Maria, Av. Roraima 1000, Santa Maria 97105-900, Brazil; bianca.maia@acad.ufsm.br (B.C.M.-d.-A.); tais.pieta@acad.ufsm.br (T.B.P.); luisa.zanon@acad.ufsm.br (L.F.Z.); gabriele.cogo@acad.ufsm.br (G.C.C.); laisa.pes@acad.ufsm.br (L.P.N.); bruna.fracari@acad.ufsm.br (B.F.d.N.); clarice.rolim@ufsm.br (C.M.B.R.); 2 Departamento de Química, Universidade Federal de Santa Maria, Av. Roraima 1000, Santa Maria 97105-900, Brazil; nayrarocha0012@gmail.com (N.S.R.); rodriguesoed@gmail.com (O.E.D.R.); 3 Laboratório de Engenharia e Processos Químicos, Universidade Federal de Santa Maria, Av. Roraima 1000, Santa Maria 97105-900, Brazil; leticiabuenomacedo@gmail.com; 4 Departamento de Físico-Química, Universidade Federal de Santa Maria, Av. Roraima 1000, Santa Maria 97105-900, Brazil; tielle.almeida@ufsm.br; 5 Departamento de Farmácia Industrial, Universidade Federal de Santa Maria, Av. Roraima 1000, Santa Maria 97105-900, Brazil; scheila.schaffazick@ufsm.br

**Keywords:** in silico studies, in vitro antitumor activity, organoselenium compound, drug combination therapy, multidrug resistance

## Abstract

**Background/Objectives:** Cancer is a major health concern involving abnormal cell growth. Combining anticancer agents can enhance efficacy and overcome resistance by targeting multiple pathways and creating synergistic effects. **Methods:** This study used in silico approaches to evaluate the physicochemical and pharmacokinetic profiles of the innovative organoselenium nucleoside analog Di3a, followed by the design of two nanocarriers. Di3a-loaded PLGA nanocapsules and nanostructured lipid carriers based on compritol were prepared and evaluated alone and combined with doxorubicin (DOX) and docetaxel (DTX) for a synergistic effect. **Results:** Di3a subtly violated some of Lipinski’s rules, but still showed suitable pharmacokinetic properties. Both nanoparticles presented nanometric size, negative zeta potential and polydispersity index values < 0.20. Hemolysis assay suggested a pH-dependent pattern conferred by the surfactant 77KL, and evidenced the biocompatibility of the formulations, aligning with the results observed in the nontumor L929 cell line. The lack of drug release studies under varying pH conditions constitutes a limitation and warrants further investigation to validate the pH-responsive properties of the nanocarriers. MTT assay revealed that both formulations exhibited significant cytotoxic effects in the A549 cell line. However, neither formulation exhibited marked toxicity toward NCI/ADR-RES, a resistant tumor cell line. Conversely, when combined with DOX or DTX, the treatments were able to sensitize these resistant cells, achieving expressive synergistic antitumor activity. **Conclusions:** Despite the limitations in the in silico studies, the study highlights the potential of combining the proposed nanocarriers with conventional antitumor drugs to sensitize multidrug-resistant cancer cells and emphasizes the safety of the developed nanoformulations.

## 1. Introduction

One of the main concerns in public health worldwide is cancer, a group of diseases characterized by abnormal and rapid cell growth [1,2]. In 2022, nearly 20 million new cases of this disease were registered globally, with lung cancer being the most common tumor diagnosed and the leading cause of cancer-related death [3]. Regarding women’s health, ovarian cancer is a highly prevalent malignancy that affects the female reproductive organs, with 324,603 new cases and 206,956 deaths recorded in 2022 [4,5]. About 90% of ovarian cancers originate from epithelial tissue and are classified into five major histotypes, each responding differently to treatment [4].

Usually, cancer treatment involves surgery, radiotherapy, and/or systemic therapy, such as chemotherapy, hormonal treatments, and targeted biological therapies, used alone or together [2]. Despite advances in surgical and chemotherapeutic options, treatment remains a challenge, mainly due to non-specific toxicity and the development of resistance to multiple drugs (MDR) [6,7]. Therefore, the search for new therapeutic strategies is extremely important and includes seeking new molecules with anticancer properties, exploring combination therapies, and developing innovative drug delivery systems [8].

Nucleoside analogs are a class of antimetabolites with antitumor properties. These molecules have structural similarity to intrinsic nucleosides, such as purine and pyrimidine, making them suitable for phosphorylation and, consequently, DNA/RNA intercalation, which can lead to disruption of metabolic and regulatory pathways [9,10]. Organoselenium compounds derived from zidovudine are an example of new nucleoside analogs that have shown potential against tumor cells [11]. However, resistance to these antimetabolites is common, mainly due to difficulties in conversion to their active metabolites and a decrease in the expression of nucleoside transporters, leading to restricted absorption [9].

The main strategy to overcome resistance and improve treatment efficiency is activating multiple downstream pathways through combining anticancer compounds, aiming for a synergistic or additive effect [12,13,14]. Furthermore, combined therapy reduces the required dosages and offers additional anticancer benefits, such as better target selectivity and decreased tumor growth and metastatic potential [14,15,16].

Developing nano-drug delivery platforms offers a promising approach to mitigate MDR in cancer cells [17]. Due to their unique properties, various nanocarriers have been explored for their ability to enable targeted delivery and enhance the permeability and retention effect (EPR), which leads to increased cell uptake by crossing physiological barriers and bypassing the efflux pump, resulting in drug accumulation in tumor tissue [18,19].

In this context, polymeric nanoparticles, specifically nanocapsules (NCs), made from biocompatible and biodegradable polymers, are versatile carriers that have been extensively studied in oncology research. Besides their advantages in overcoming the MDR effect, these systems also offer benefits such as improving stability and bioavailability of molecules, reducing systemic side effects, enabling controlled release, and allowing surface modification [20,21].

Lipid-based nanoparticles are another category explored as potential drug carriers, including nanostructured lipid carriers (NLCs), developed as an improvement on liposomes and solid lipid nanoparticle systems. The NLC formulation consists of a core matrix loaded with both solid and liquid lipids, which allows reduction in drug leakage, optimization of drug delivery, and improvement of stability and loading capacity for lipophilic molecules, while maintaining protection function, biocompatibility, and non-immunogenicity [21,22]. Furthermore, the NLCs’ characteristics, such as physiological, biodegradable, and biocompatible lipid materials and surfactants, make them suitable for regulatory acceptance as drug delivery systems [23].

Facing the limitations of cancer monotherapy, the development of resistance to multiple drugs, and considering the potentialities of nanosystems, we proposed a new organoselenium compound derived from zidovudine, 5′-Seleno-(phenyl)-(3-N)-5′-Seleno-(4-methylphenyl)-4-N(6-chloro-1,3,5-triazin-2-yl)-thymidine (Di3a), as an innovative compound for cancer therapy. First, we predicted its physicochemical and pharmacokinetics properties using in silico platforms. We also evaluated the in vitro antitumor potential of the molecule, using the cell lines A549 (human lung adenocarcinoma) and NCI/ADR-RES (multidrug-resistant human ovarian carcinoma). In order to achieve a targeted treatment, we designed two nanocarriers. We used poly(lactic-co-glycolic acid) (PLGA) to obtain polymeric NPs, and Compritol^®^ 888 and medium chain triglycerides (MCT) to obtain NLCs. Both nanocarriers incorporated poloxamer 407, a stabilizer surfactant that increases tumor sensitization, and 77KL, a pH-dependent surfactant [24]. The optimized nanoformulations were assessed regarding their physicochemical characteristics. The effect of pH on the membrane-lytic activity of the nanocarriers was assessed using erythrocytes as models for endosomal membranes. Additionally, the nanocarriers’ safety was tested through a hemocompatibility assay, and their non-specific cytotoxicity was examined using a non-tumor cell line (L929). Their possible antitumor effects were evaluated with both sensitive (A549) and resistant/MDR (NCI/ADR-RES) tumor cell lines.

Finally, considering previous data indicating that selenium-derived compounds exhibit synergistic effects with various cancer treatments, along with the benefits of combination therapy, we investigated the NPs and NLCs synergic antitumor activity with doxorubicin (DOX) and docetaxel (DTX), two antineoplastic drugs widely applied in the treatment of different types of cancer [25,26,27].

## 2. Materials and Methods

### 2.1. Materials

HPLC-grade methanol and acetonitrile from Sigma-Aldrich (São Paulo, SP, Brazil), and acetone from Dinâmica (São Paulo, SP, Brazil). The polymer Poly(D, L-lactic-co-glycolic acid) (PLGA, 50:50, 24–38 kDa), Poly(vinyl alcohol) (average mol wt 30,000–70,000), sorbitan monooleate (Span80^®^, molar mass = 428 g/mol), poloxamer 407 (Pluronic^®^ F-127; molar mass = 12,600 g/mol, 70% EO content), 2,5-diphenyl-3-(4,5-dimethyl-2-thiazolyl) tetrazolium bromide (MTT), trypsin-EDTA solution, antibiotic solution of penicillin/streptomycin, fetal bovine serum (FBS) and Dulbecco’s Modified Eagle’s Medium (DMEM), supplemented with L-glutamine (584 mg/L) were obtained from Sigma–Aldrich (São Paulo, SP, Brazil). Dimethyl sulfoxide (DMSO) was obtained from Neon (Suzano, SP, Brazil), and Compritol^®^ 888 ATO (melting point: 65–77 °C; hydroxyl value: ≤5 mg KOH/g; fatty acid composition: behenic acid ≥ 83%, arachidic acid ≤ 10% stearic acid ≤ 5%, palmitic acid ≤ 3%, lignoceric acid ≤ 3% and erucic acid ≤ 1%) was kindly donated by Gattefossé España S.A. (Barcelona, Spain). Zibo Ocean International Trade (Zibo, China) supplied doxorubicin (DOX) and docetaxel (DTX).

The organoselenium compound Di3a was synthesized and fully characterized as previously described [28] by the LabSelen-NanoBio at the Federal University of Santa Maria (Santa Maria, Brazil). The surfactant 77KL (molar mass = 405 g/mol, purity ~98% and critical micellar concentration = 2.9 × 10^3^ µg/mL), used as a potential pH-responsive adjuvant in the nanoformulations, is derived from the amino acid lysine (N^α^,N^ε^-dioctanoyl lysine, 77K) and includes an inorganic lithium counterion [24]. It was generously provided by Consejo Superior de Investigaciones Científicas (Barcelona, Spain).

### 2.2. Procedure for the Synthesis of 5′-Seleno-(phenyl)-(3-N)-5′-Seleno-(4-methylphenyl)-4-N(6-chloro-1,3,5-triazin-2-yl)-thymidine

In a 50 mL round-bottom flask equipped with a reflux condenser, 0.2 mmol of 5′-Seleno-(phenyl)-3-(amino)-thymidine, 5 mL of acetone, and 0.2 mmol of 5′-Seleno-(4-methyl)-(3-N-4,6-dichloro-1,3,5-triazin-2-yl)-thymidine dissolved in 6 mL of acetone were added. After 5 min of reaction, 0.2 mmol of N, N-diisopropylethylamine (DIPEA) was added. The reaction mixture was then allowed to proceed for 24 h at 55 °C under reflux. Reaction progress was monitored by thin-layer chromatography (TLC). After completion, the product was extracted with dichloromethane (3 × 20 mL) and a mixture of distilled water, NaCl, and HCl (approximately 60 mL). The organic layer was dried over anhydrous MgSO_4_, filtered, and the solvent was evaporated to afford the crude product. The crude material was purified by column chromatography using silica gel and a dichloromethane: ethanol (97:3) eluent system [28].

### 2.3. In Silico Prediction of Physicochemical Properties and Pharmacokinetics Study

PubChem (https://pubchem.ncbi.nlm.nih.gov/, accessed on 11 March 2025) is an open chemistry database maintained by the National Institutes of Health (NIH). The database supplied the chemical structure and SMILES for Di3a, which were then used in subsequent analyses.

The Molinspiration (https://www.molinspiration.com/, accessed on 11 March 2025) platform was used to assess the compound according to Lipinski’s rules, describing its physicochemical properties and establishing criteria that indicate the potential for successful oral bioavailability of a new compound.

The pkCSM (https://biosig.lab.uq.edu.au/pkcsm/, accessed on 11 March 2025) and SwissADME (http://www.swissadme.ch/, accessed on 11 March 2025) platforms were utilized to forecast the pharmacokinetic properties of the compound Di3a and Lipinski’s rules. Additionally, its toxic potential was assessed using the ProTox-3.0 platform (https://tox.charite.de/protox3/, accessed on 11 March 2025).

### 2.4. Analytical Method

High-performance liquid chromatography (HPLC) method validation was conducted following international guidelines, encompassing specificity, accuracy, linearity, precision, and robustness [29]. Chromatographic separation was performed under isocratic conditions at room temperature using a Shimadzu LC system (Shimadzu, Kyoto, Japan) paired with an SPD-M20A photodiode array (PDA) detector (Shimadzu, Kyoto, Japan). The analysis utilized a Kinetex EVO C18 column (Phenomenex, Torrance, CA, USA; 150 mm × 4.6 mm, 5 µm) with UV detection set at 263 nm. The mobile phase consisted of acetonitrile, potassium phosphate buffer (15 mM, pH 3.0), and methanol in a 45:40:15 (*v*/*v*/*v*) ratio, delivered at a flow rate of 1.0 mL/min. This reverse-phase LC (RP-LC) method was designed explicitly for quantifying Di3a in both formulations.

### 2.5. Nanoparticles Preparation

Polymeric and lipid-based nanoparticles encapsulating Di3a were prepared using nanoprecipitation [30] and spontaneous emulsification methods [31], respectively.

The NC organic phase was prepared by dissolving 40 mg of sorbitan monooleate, 7.5 mg of TCM, and 25 mg of PLGA in 30 mL of acetone, followed by magnetic stirring at 300 rpm for 20 min. 15 mg of Di3a was dissolved in 2 mL of a 1:20 (*v*/*v*) mixture of DMSO and acetone and added to the organic solution. Simultaneously, a 50 mL aqueous solution was prepared by dissolving 120 mg of poloxamer 407, then adding 2.5 mg of the surfactant 77KL.

The organic phase for the NLC was obtained by solubilizing 40 mg of sorbitan monooleate, 15 mg of TCM, and 35 mg of Compritol in 30 mL of acetone, and subsequently subjected to magnetic stirring at 300 rpm for 20 min at 65 °C. Di3a was dissolved in 2 mL of a DMSO: acetone mixture (1:20, *v*/*v*) and then added to the organic phase. Meanwhile, the aqueous phase was prepared by dissolving 150 mg of poloxamer 407 in 50 mL of water, followed by the addition of 60 mg of PVA, and finally 2.5 mg of the surfactant 77KL.

In both formulations, the organic phase was slowly added to the aqueous phase under vigorous stirring at 850 rpm, and the mixture was stirred for 10 min. The formulations were then concentrated under reduced pressure until the final volume reached 5 mL (3 mg/mL of Di3a), resulting in the Dia3a-77KL-NC or Di3a-77KL-NLC nanoparticle suspensions. For comparative purposes, the same volume of formulation was utilized for the NPs without Di3a-loaded NPs.

### 2.6. Nanoparticles Characterization

Dynamic light scattering (DLS) analyses were performed using a Malvern Zetasizer ZS (Malvern Instruments, Malvern, UK) to determine the hydrodynamic diameter and polydispersity index (PDI) of the NPs. Before analysis, all samples were diluted in ultrapure water at a 1:500 (*v*/*v*) ratio. Zeta potential (ZP) was also measured with the same instrument by assessing the electrophoretic mobility of the formulations diluted in 10 mM NaCl aqueous solution (1:500, *v*/*v*). Finally, the pH of NP suspensions was measured at room temperature using a calibrated pH meter (Orion 3 Star, Thermo Scientific, Waltham, MA, USA). Each analysis was performed in triplicate.

The HPLC method was used to measure active compound content and encapsulation efficiency (EE%). To determine the active compound content in nanoformulations, samples were first diluted in acetonitrile (1:10, *v*/*v*) and vortexed for 10 min at 1300 rpm. The NC suspensions were then sonicated for 10 min at 40 °C, and NLC suspensions were centrifuged at 8000 rpm for 5 min. Afterwards, the samples were further diluted in the mobile phase to a final concentration of 25 µg/mL, filtered through a 0.45 µm membrane, and injected into the RP-LC system. To assess the total active compound content in the formulations, the Di3a reference solution was diluted in the mobile phase to achieve an equivalent concentration.

The EE% of the bioactive compound was evaluated using the ultrafiltration/centrifugation method with Amicon Ultra 0.5 centrifugal filters (10,000 Da MWCO, Millipore, Burlington, VT, USA). For this, 300 µL of NC or NLC nanoparticles were placed into the device and centrifuged at 3610× *g* for 20 min. Ultrafiltrate analysis, representing the non-encapsulated fraction, was performed using RP-LC. Drug loading (DL%) was calculated by dividing the amount of Di3a encapsulated by the total NP mass, including excipients, and then expressed as a percentage. The EE% and DL% were then determined using the following equations:

$$EE\%=\left(\frac{total\;content-free\;content}{total\;content}\right)\times 100$$

$$DL\%=\left(\frac{mass\;of\;drug\;in\;NPs(mg)}{total\;mass\;of\;NPs(mg)}\right)\times 100$$

The size and morphology of the NC and NLC were assessed using atomic force microscopy (AFM) (Park Systems, Suwon, Republic of Korea). For AFM imaging, 2 µL of NPs was placed on freshly cleaved mica substrates and allowed to dry at room temperature. Scanning was performed in non-contact mode. Topographical images were captured with a Park NX10 microscope (Park Systems, Suwon, Republic of Korea) using Smart Scan software (version 1.0 RTM11a). Measurements utilized a highly doped silicon monolithic probe with a reflective aluminum coating (PPP-NCHR-50, Nanosensors, Neuchatel, Switzerland), featuring a nominal resonance frequency between 204 and 497 kHz and a force constant of 10 to 130 N/m. All tests were conducted under controlled environmental conditions (22 ± 2 °C and 55 ± 10% relative humidity). Image analysis was performed using XEI software (version 4.3.4 Build 22, RTM1) and ImageJ, version 1.51.

### 2.7. Nanoparticles Stability Study

To assess the stability of the NPs, physicochemical properties, including mean hydrodynamic diameter, PDI, pH, and ZP and Di3a content, were monitored over 30 days. The polymeric NPs were stored at room temperature (22 °C ± 2 °C), while the lipidic NPs were kept refrigerated (5 °C ± 3 °C). All measurements were performed in triplicate.

### 2.8. Hemocompatibility Studies

Blood compatibility was evaluated through a hemolysis assay, as previously described [32]. Erythrocytes were collected from healthy human volunteers, in accordance with the ethical guidelines approved by the Research Ethics Committee of the Federal University of Santa Maria, Brazil (protocol CAAE 86843725.3.0000.5346). After centrifugation, the red blood cells were washed twice and resuspended in isotonic phosphate-buffered saline (PBS, pH 7.4; 300 mOsm/L) at a final concentration of 8 × 10^9^ cells/mL. The tested formulations included Di3a-77KL-NC, 77KL-NC, Di3a-77KL-NLC, 77KL-NLC, and non-encapsulated Di3a, at concentrations of 150, 200, and 250 µg/mL. Each sample was incubated with 25 µL of erythrocyte suspension under gentle agitation for 5 h. The reaction was halted by centrifuging at 10,000× *g* rpm for 5 min. Positive and negative controls were established by adding 25 µL of erythrocyte suspension to distilled water or PBS, respectively. After centrifugation, 200 µL of each supernatant was transferred to a 96-well plate, and hemolysis was measured by absorbance at 550 nm using a microplate reader (Multiskan FC, Thermo Fisher Scientific, Shanghai, China). For comparison, the same formulation volume was used for NPs unloaded Di3a.

### 2.9. pH-Dependent Membrane-Lytic Activity of Nanoparticles

To investigate the potential pH-dependent membrane-lytic activity of the nanoformulations, a protocol similar to the hemocompatibility test was used, using erythrocytes as models for the endosomal membrane [33]. The NPs suspensions (Di3a-77KL-NC and Di3a-77KL-NLC) were diluted in PBS at pH levels of 7.4, 6.6, or 5.4 to final concentrations of 250, 200, or 150 µg/mL, respectively. They were then incubated with 25 µL of erythrocyte suspension under gentle agitation for 5 h at room temperature. Positive controls were prepared by dispersing erythrocytes in water, whereas negative controls were prepared with PBS. The reaction was halted by centrifuging at 10,000× *g* rpm for 5 min, and absorbance was measured at 550 nm with a 96-well microplate reader (Multiskan FC, Thermo Fisher Scientific, Shanghai, China). For comparative purposes, control NPs were also prepared, including 77KL-NC and 77KL-NLC (without the active compound), as well as Di3a-NC, NC, Di3a-NLC, and NLC (without the pH-sensitive adjuvant 77KL).

### 2.10. In Vitro Protein Corona

Nanoformulations were diluted in DMEM supplemented with 5% FBS or in human plasma at a final concentration of 100 µg/mL. The human plasma was obtained from healthy human volunteers, in accordance with the ethical guidelines approved by the Research Ethics Committee of the Federal University of Santa Maria, Brazil (protocol CAAE 86843725.3.0000.5346). The mean hydrodynamic diameter and PDI were measured using a Zetasizer ZS (Malvern Instruments, Malvern, UK) at two time points: immediately after dilution (t = 0 h), and after 72 h of incubation at 37 °C, simulating conditions during in vitro cytotoxicity tests and potential in vivo exposure. As a control, the particle size and PDI of the NPs in aqueous suspension was measured under similar conditions [34].

### 2.11. In Vitro Cell Biocompatibility Studies

To evaluate nonspecific cytotoxicity, the L929 cell line (murine fibroblasts) was used. These non-tumor cells were obtained from the Rio de Janeiro cell bank (BCRJ, Rio de Janeiro, Brazil). The cells were cultured in DMEM (4.5 g/L glucose) supplemented with 10% (*v*/*v*) fetal bovine serum (FBS), under standard conditions (37 °C, 5% CO_2_). Once they reached approximately 80% confluence, the cells were harvested and then seeded into 96-well plates at a density of 1.0 × 10^5^ cells/mL, followed by incubation for 24 h. Then, they were exposed to non-encapsulated and Di3a-loaded nanosystems for 24 h under the same culture conditions. The MTT assay was used as an endpoint to evaluate cell viability, and absorbance was measured at 550 nm using a Multiskan FC microplate reader (Thermo Fisher Scientific, Shanghai, China). The negative control (cells exposed to treatment-free medium) was taken as 100% cell viability. The cytotoxicity was also expressed in terms of IC_50_ (concentration causing 50% death of the cell population), calculated from concentration-response curves.

### 2.12. In Vitro Antitumor Activity

The A549 (human lung cancer) cell line and the MDR human ovarian carcinoma cell line, NCI/ADR-RES, were used in this study. The A549 cell line was obtained from the Rio de Janeiro cell bank (BCRJ, Rio de Janeiro, Brazil), and the NCI/ADR-RES cell line was kindly donated by the University of Girona (Girona, Spain). The cells were maintained in complete DMEM (4.5 g/L glucose), supplemented with 10% (*v*/*v*) fetal bovine serum (FBS). For NCI/ADR-RES cells, the medium was also supplemented with 1.0 µg/mL of DOX. Cells were cultured in 75 cm^2^ flasks at 37 °C in a humidified incubator with 5% CO_2_. Cultures were maintained until they reached approximately 80% confluence, then the cells were harvested, seeded into 96-well plates, and incubated for 24 h under the same conditions.

Both cell lines were used to assess the potential in vitro antiproliferative effects of Di3a in its non-encapsulated and nanoencapsulated forms: A549 (2.5 × 10^4^ cells/mL) and NCI/ADR-RES (1 × 10^5^ cells/mL). After incubation, the medium was replaced with 100 µL of treatment solutions prepared in DMEM supplemented with 5% FBS. Non-encapsulated Di3a, Di3a-77KL-NC, and Di3a-77KL-NLC were administered at concentrations of 5, 25, 50, 75, and 100 µg/mL. Nanoformulations without Di3a were also diluted at the same proportion NP/medium to evaluate any influence of the systems. Control cells received only DMEM with 5% FBS. Cells were exposed to the treatments for 72 h, followed by a 3 h incubation at 37 °C in a 5% CO_2_ atmosphere with 0.5 mg/mL MTT solution prepared in DMEM. After incubation, the medium was removed and replaced with 100 µL DMSO to solubilize the formazan crystals. Using a microplate reader, absorbance was subsequently measured at 550 nm. (Multiskan FC, Thermo Fisher Scientific, Shanghai, China). The negative control was taken as 100% cell viability and the cytotoxicity of each treatment was also expressed in terms of its IC_50_, calculated from concentration-response curves.

### 2.13. Synergic In Vitro Antitumor Activity

To evaluate the synergistic effect of DOX or DTX in combination with NPs, MDR human ovarian cancer cells (NCI/ADR-RES cell line) were used. The cells were cultured and maintained as described in Section 2.12. After seeding in 96-well plates (1 × 10^5^ cells/mL), they were incubated for 24 h at 37 °C in a humidified atmosphere containing 5% CO_2_ before treatment. Treatments included non-encapsulated and nanoencapsulated Di3a, and combinations with DOX or DTX, to evaluate the effects of each bioactive compound, the influence of nanoformulations, and potential synergistic interactions. The NPs’ concentrations tested ranged from 5 to 100 µg/mL; DOX and DTX were used at 10 and 0.5 µg/mL, respectively. After 72 h, cells were incubated with 0.5 mg/mL MTT in DMEM for 3 h at 37 °C with 5% CO_2_. The MTT solution was then replaced with 100 µL DMSO, and absorbance was measured at 550 nm using a Thermo Fisher Scientific Multiskan FC microplate reader in Shanghai, China. The combination index (CI) was calculated using the median-effect method with CompuSyn software (version 1.0). According to the Chou–Talalay method, CI values < 0.9 indicate a synergistic effect, 0.9–1.1 suggest an additive effect, and values > 1.1 denote antagonism [35,36].

### 2.14. Statistics

All data are presented as mean ± standard error (SE) or mean ± standard deviation (SD). Statistical analysis was performed using one-way analysis of variance (ANOVA) to identify significant differences among groups, followed by Tukey’s post hoc tests for multiple comparisons. The studies were conducted using SPSS software (version 22; SPSS Inc., Chicago, IL, USA). The robustness of the RP-LC method was assessed using Minitab^®^ Statistical Software (Release 17; Minitab Inc., State College, PA, USA). The IC_50_ values were determined using GraphPad Prism version 8. All experiments were conducted in triplicate, and statistical significance was set at a confidence level of *p* < 0.05 or *p* < 0.01.

## 3. Results

### 3.1. Synthesis of Di3a

**Figure 1:**
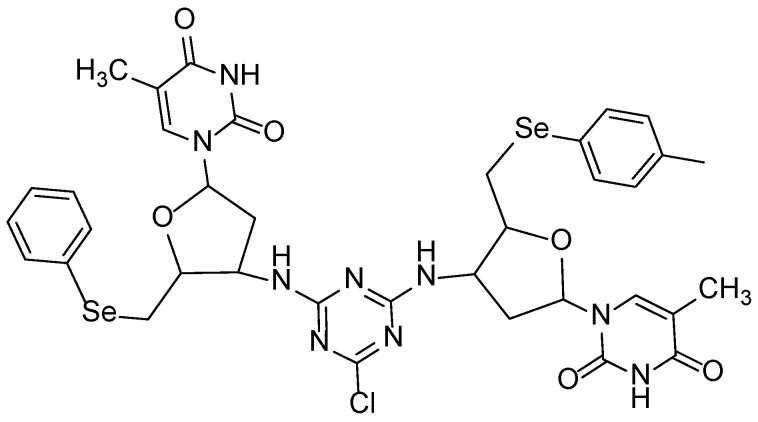
Di3a chemical structure.

The organoselenium Di3a was successfully synthesized with the molecular formula C_36_H_38_ClN_9_O_6_Se_2_ and a molecular weight of 886 g·mol^−1^, with the process yielding the final product in a highly efficient 92% yield (Figure 1).

### 3.2. In Silico ADMET and Physicochemical Properties Assessments 

SMILE representation was derived from the molecule’s chemical structure, as per the PubChem database 7ccc([Se]CC2OC(n1cc(C)c(=O)[nH]c1=O)CC2Nc6nc(Cl)nc(NC4CC(n3cc(C)c(=O)[nH]c3=O)OC4C[Se]c5ccccc5)n6)cc7, facilitating subsequent computational analyses.

Using the Molinspiration, pKCSM, and SwissADME platforms, we evaluated the molecule’s physicochemical properties. Despite some differences between these platforms, the average values showed a logP of 5.45, indicating lipophilic behavior, along with a high polar surface area (TPSA) of 191 Å. The molar mass (886 g/mol), 12 hydrogen bond acceptors, 4 donors, and 12 rotatable bonds contribute to the molecule’s high structural complexity. Additionally, we observed three violations of Lipinski’s rules, which suggest low permeability in biological membranes and may limit oral bioavailability (Table 1).

According to the platforms, Di3a may or may not be a substrate and inhibitor of P-glycoprotein (P-gp), and it has a low ability to cross the blood–brain barrier (BBB). Its predicted plasma protein binding is very high, indicating that most of the compound will stay bound in circulation. Additionally, the expected volume of distribution is low, suggesting a primarily intravascular distribution.

Di3a’s metabolism is linked to the CYP3A4 enzyme. According to pkCSM, admetSAR, and SwissADME, it is a CYP3A4 substrate and inhibitor. Additionally, SwissADME confirms that Di3a inhibits CYP3A4. In contrast, other cytochromes, including CYP1A2, CYP2C19, CYP2C9, and CYP2D6, are not significantly affected.

Regarding excretion, pkCSM predicts a low total clearance of −0.7 log ml/min/kg, indicating that Di3a is not a substrate of renal OCT2 transporters. This suggests that elimination is likely to be slow and not strongly influenced by renal secretion.

Most toxicity prediction tools agree that Di3a is not mutagenic or carcinogenic, as indicated by negative AMES test results. However, the ProTox platform suggests a high likelihood of hepatotoxicity, neurotoxicity, and nephrotoxicity. pkCSM also identifies hERG II inhibition, which is linked to cardiac arrhythmias, but not hERG I. Table 2 presents a summary of the data collected.

### 3.3. Physicochemical Characterization of Nanoformulations 

The nanoformulations were characterized based on their physicochemical properties, as summarized in Table 3, which includes measurements of mean hydrodynamic diameter, pH, ZP, PDI, Di3a content, EE%, and DL%. After the incorporation of Di3a, there was a slight increase in size in both formulations (from 116 nm to 120 nm in the polymeric, and from 217 nm to 259 nm in the lipidic). Both formulations showed very high EE%, nearly 99.9%, indicating Di3a’s strong affinity for the different matrices. The polymeric matrix achieved a greater DL of 7.2%, whereas the lipid NP had a lower DL of 4%.

The NP suspensions showed nanoscale sizes with a narrow size distribution, as indicated by low PDI values. They also had a negative surface charge (ZP) and a slightly acidic pH level. Table S1 presents the complete data of NPs physicochemical characterization.

**Figure 2:**
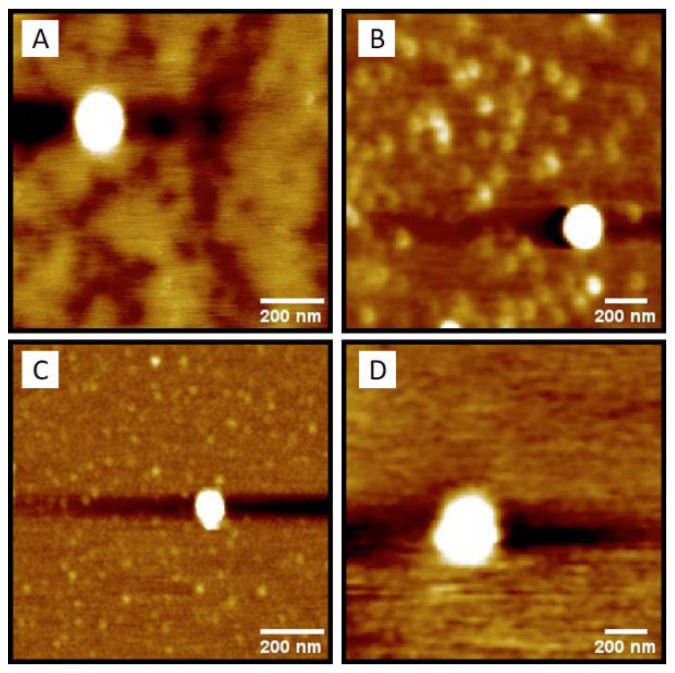
Nanoparticle’s morphology observed by AFM: (**A**) 77KL-NC, (**B**) Di3a-77KL-NC, (**C**) 77KL-NLC, and (**D**) Di3a-77KL-NLC.

Additionally, AFM analysis showed that the NPs had a spherical shape with a uniform surface (Figure 2).

### 3.4. Stability Profile of Nanoparticles

The 30-day stability profile of the nanoformulations is summarized in Table S2. All polymeric NPs and Di3a-loaded lipidic NPs maintained a stable mean particle size, whereas unloaded lipidic NPs exhibited a moderate time-dependent increase in mean diameter. Only the Di3a-77KL-NLC did not show PDI < 0.2 at 30 days; the others maintained PDI below 0.2 over time, with the polymeric ones exhibiting lower and more stable values (~0.08–0.14), indicating homogeneity of the systems. The zeta potential of the NPs was negative, ranging from approximately −2 to −8, and the pH of the polymeric NPs was slightly more acidic than that of the lipidic ones. The Di3a content showed a slight decline after 30-day storage, from 3.06 mg/mL to 2.84 mg/mL in Di3a-77KL-NC, and from 2.5 mg/mL to 2.37 mg/mL in Di3a-77KL-NLC.

### 3.5. Analytical Method Development and Validation

The validation of the RP-LC method confirmed its robustness, as minor variations in flow rate, organic solvent content, and injection volume (evaluated using a three-factor, two-level factorial design) did not significantly impact the assay results (*p* > 0.05), as shown by the Pareto chart (Figure S1). Method specificity was demonstrated by peak purity analysis using a PDA detector; the Di3a peak showed a purity index above 0.9999, indicating no co-eluting interference (Figures S2 and S3). Accuracy was verified by recovery values ranging from 98% to 102% (Table S3). Precision was confirmed by relative standard deviation values below the 2% acceptance limit for repeatability, inter-day, and between-analyst assessments (Table S4). Additionally, the method displayed excellent linearity across the concentration range of 10–60 µg/mL (y = 38,652x + 41,647, r = 0.9999), with significant linear regression (Fcalc = 14,878.76 > Fcritical = 4.75, *p* < 0.05) and no deviation from linearity (Fcalc = 0.41 < Fcritical = 3.26, *p* > 0.05) (Figure S4).

### 3.6. Hemocompatibility Assessment 

**Figure 3:**
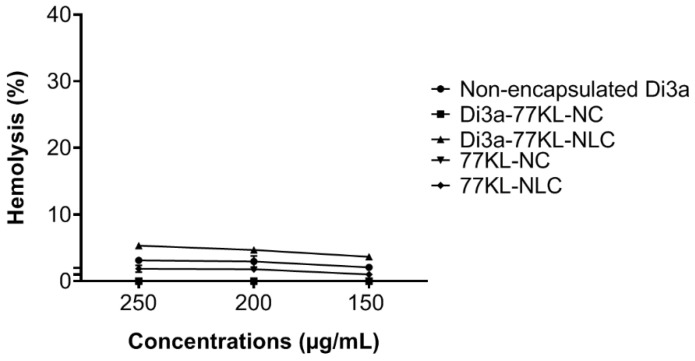
Hemocompatibility study performed after 5 h of incubation with human erythrocytes. The results are expressed as mean ± SE.

Assessing blood compatibility is crucial when developing NPs systems for systemic use, as it provides important insights into their hemocompatibility and safety. All tested formulations showed little to no hemolytic activity. Notably, polymeric NPs—both Di3a-77KL-NC and 77KL-NC—had near-zero hemolysis, indicating compatibility with erythrocytes. Similarly, non-encapsulated Di3a and the nanostructured lipid carrier 77KL-NLC exhibited minimal hemolysis (~3 and 2%, respectively). A small hemolytic effect (5.34%) was only observed in lipid carriers loaded with Di3a at the highest concentration, suggesting a concentration-dependent response in this case (Figure 3).

### 3.7. pH-Responsive Membrane Disruptive Activity of Nanoparticles 

**Figure 4:**
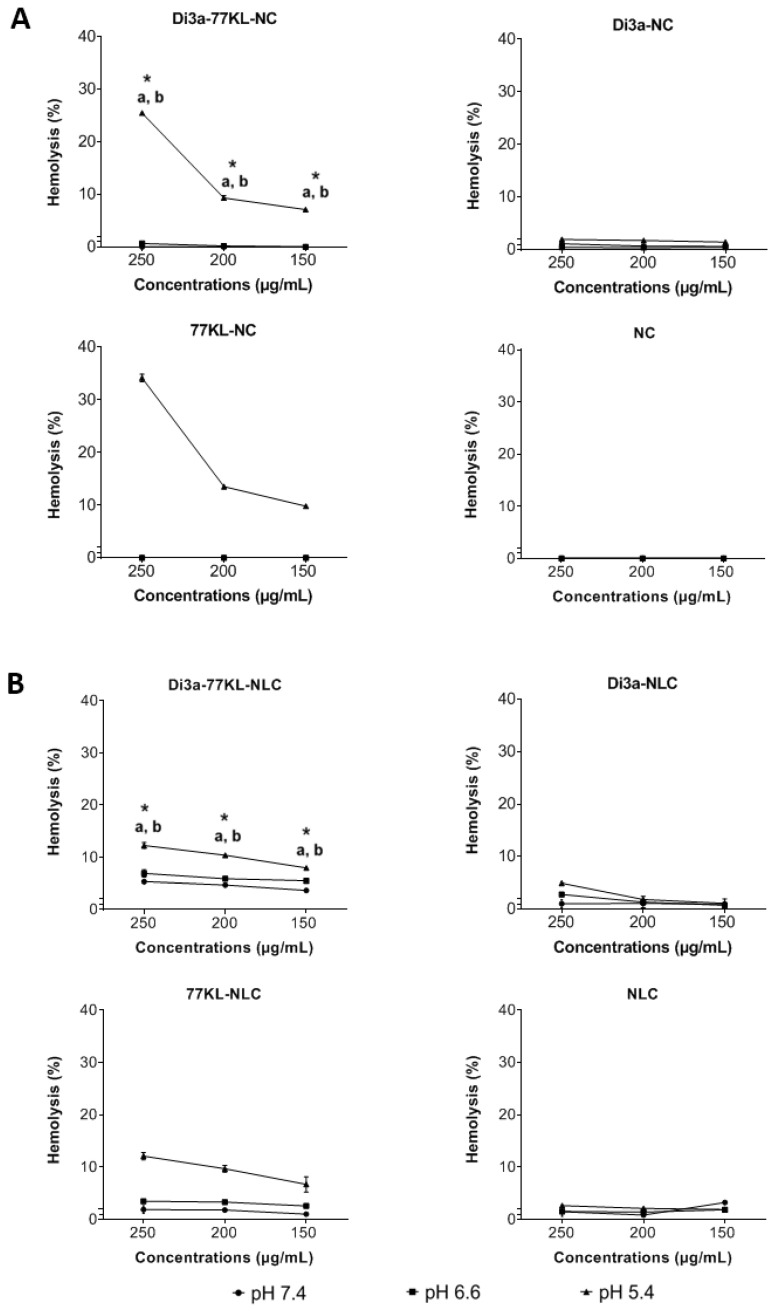
pH-dependent hemolytic activity of (**A**) polymeric, and (**B**) lipidic NPs after 5 h of incubation with human erythrocytes. ANOVA followed by Tukey’s test. a *p* < 0.05 vs. pH 7.4; b *p* < 0.05 vs. pH 6.6; * indicates *p* < 0.05 between NPs with and without.

At a physiological pH, the tested nanocarriers showed minimal interaction with red blood cells, with hemolysis values below 5%, except for Di3a-77KL-NLC at a 250 µg/mL concentration (~5.3%). The highest hemolysis percentages were observed for Di3a-77KL-NC (25.47%) and Di3a-77KL-NLC (12.23%) at pH 5.4 at 250 µg/mL, with a significant difference between pH 7.4 and pH 6.6 at all tested concentrations (*p* < 0.05). Unloaded NPs containing 77KL also exhibited marked hemolysis at acidic pH 5.4, with rates of 34.15% for 77KL-NC and 12.11% for 77KL-NLC, while NC, NLC, Di3a-NC, and Di3a-NLC, without the surfactant 77KL, showed minimal hemolysis (Figure 4).

### 3.8. In Vitro Protein Adsorption Studies 

**Figure 5:**
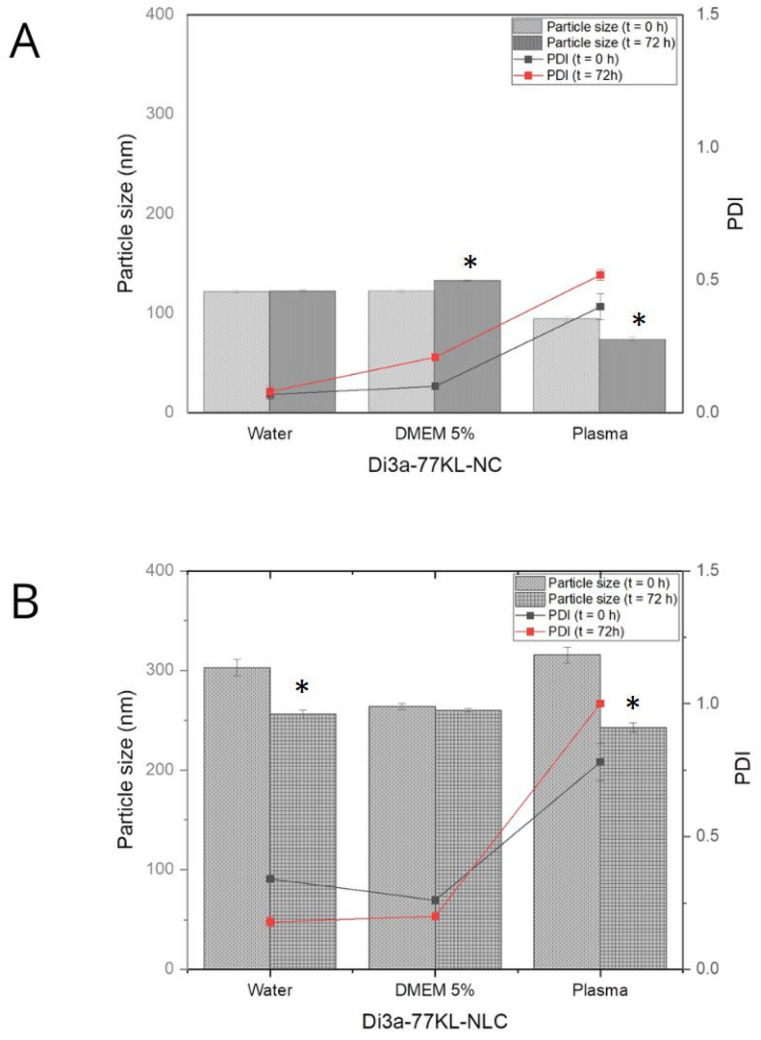
The mean particle size of nanoformulations and PDI was measured immediately after dilution (t = 0 h) and after 72 h of incubation in water, DMEM with 5% FBS, and human plasma for (**A**) Di3a-77KL-NC and (**B**) Di3a-77KL-NLC. Results are shown as mean ± SD from three independent experiments. Statistical analysis using Tukey’s test indicated significant differences between time 0 and 72 h, denoted by * (*p* < 0.05).

Figure 5 illustrates that, after 72 h of incubation—particularly in plasma media—hydrodynamic diameter values decreased. Specifically, Di3a-77KL-NC dropped from 93 nm to 73 nm, and Di3a-77KL-CLN from 315 nm to 243 nm. Human plasma initially produced the largest sizes due to its high protein content. Conversely, DMEM 5% FBS showed slight increases, while water had little impact on particle sizes, except for Di3a-77KL-NLC, which decreased from 302.73 nm to 256.07 nm. The different patterns of size reduction in polymeric and lipid formulations emphasize how the NP matrix composition affects corona stability over time. The PDI values showed no statistically significant difference between 0 and 72 h for NPs. The highest PDI values were found in plasma for both formulations. The polymeric NPs had lower PDI values compared to the lipid NPs, indicating a more uniform size distribution in the media studied.

### 3.9. In Vitro Evaluation of Cell Biocompatibility 

**Figure 6:**
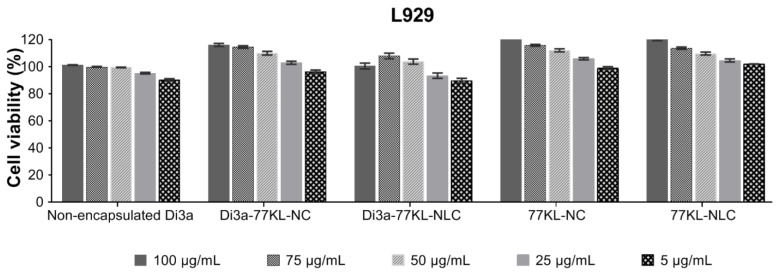
In vitro biocompatibility of NPs assessed by MTT assay using the non-tumor L929 fibroblast cell line. Results are expressed as mean ± SE from three independent experiments.

After 24 h, all treatments were non-cytotoxic to the L929 cell line, with higher cell viability observed in encapsulated formulations compared to non-encapsulated Di3a (cell viability ranging from 116.2% to 89.5%). The unloaded carriers also promoted fibroblast proliferation at the highest concentrations (cell viability ranged from 123.4% to 109.7%), confirming their biocompatibility (Figure 6). Likewise, the IC_50_ values were greater than 100 µg/mL, corroborating the low cytotoxicity of all treatments toward the non-tumor cells.

### 3.10. In Vitro Study of Anticancer Activity 

**Figure 7:**
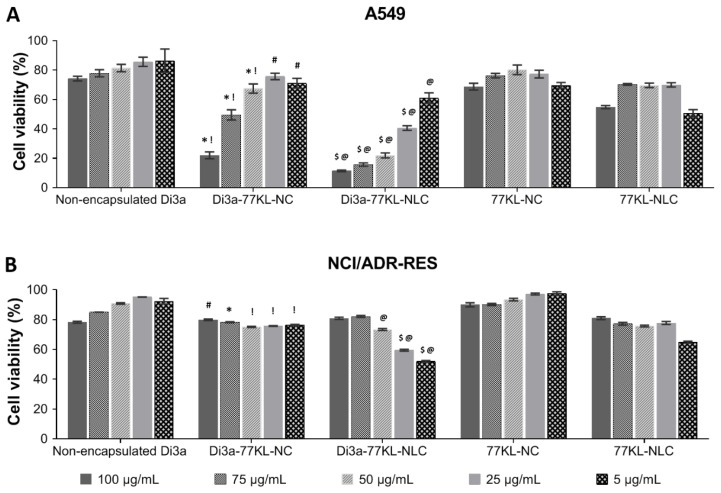
In vitro antiproliferative effects assessed by MTT assay after 72 h of treatment in (**A**) A549, and (**B**) NCI/ADR-RES cell lines. Tukey’s test showed significant differences: (**A**) * and $ indicate differences from unloaded NPs, *p* < 0.01; ! and @ denote differences from non-encapsulated Di3a, *p* < 0.01, # indicates difference from non-encapsulated Di3a, *p* < 0.05. (**B**) * and $ indicate differences from unloaded NPs, *p* < 0.01; ! and @ denote differences from non-encapsulated Di3a, *p* < 0.01, # indicates difference from non-encapsulated Di3a, *p* < 0.05.

The viability of A549 cells after 72 h of exposure to various Di3a-based formulations was assessed with the MTT assay (Figure 7A). Non-encapsulated Di3a demonstrated the highest cell viability, indicating minimal cytotoxicity, with values ranging from 86.3% to 74.25%. Conversely, Di3a-77KL-NC and Di3a-77KL-NLC significantly reduced cell viability in a concentration-dependent manner, from 71% to 22% and 60.9% to 11.5%, respectively, suggesting that nanoencapsulation enhanced cytotoxic effects. The control formulations without the active compound (77KL-NC and 77KL-NLC) exhibited high cell viability, confirming the biocompatibility of the carrier systems.

On the other hand, the effects of the tested formulations in NCI/ADR-RES cells were less pronounced than in the sensitive tumor cells, as cell viability remained relatively high across all conditions and concentrations (Figure 7B). A slight to moderate decline in viability rates was noted comparing the highest (100 µg/mL) and the lowest (5 µg/mL) tested concentrations for Di3a-77KL-NC (from 79% to 76.3%) and, more markedly, for Di3a-77KL-NLC (from 80.9% to 51.9%). Table 4 presents the IC_50_ values of all treatments across both tumor cell lines. Despite the moderate reduction in cell viability induced by the nanoformulations in MDR cells, none of the tested concentrations reduced viability below 50%, thereby justifying IC_50_ values above 100 µg/mL.

### 3.11. In Vitro Antitumor Activity of Combined Treatments 

**Figure 8:**
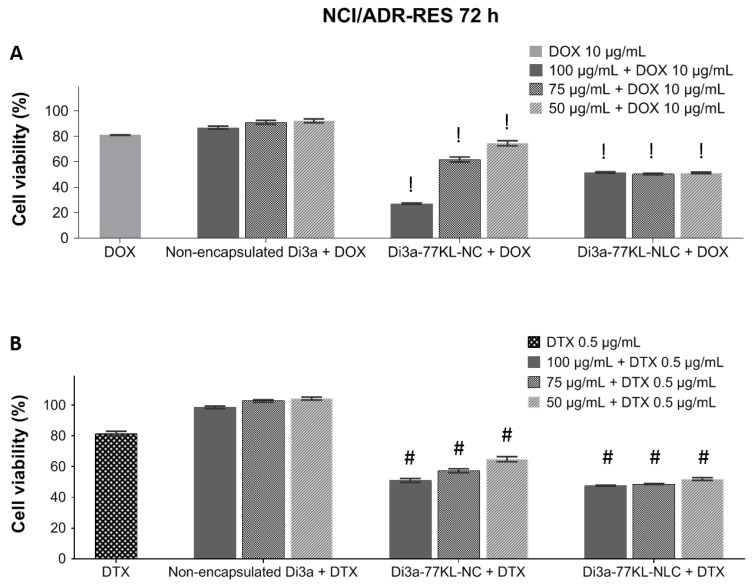
In vitro cell viability is measured by MTT after 72 h of co-incubation with either non-encapsulated or nanoencapsulated Di3a, combined with (**A**) DOX and (**B**) DTX in the NCI/ADR-RES cell line. Data represent the mean of three independent experiments ± SE. Statistical analysis was performed using ANOVA followed by Tukey’s post hoc test. ! and # indicate a significant difference from non-encapsulated Di3a + DOX or non-encapsulated Di3a + DTX, respectively (*p* < 0.01).

The synergistic cytotoxicity profiles of the NPs combined with DOX and DTX in the MDR cell line, NCI/ADR-RES, are shown in Figure 8. The concentrations of DOX and DTX used to assess the synergistic effect were optimized to keep cell viability around 80% (10 µg/mL and 0.5 µg/mL, respectively).

When combined with DOX, non-encapsulated Di3a maintained high cell viability, indicating minimal additional cytotoxicity and antagonistic effect (cell viability 87–92%, CI > 1.1). In contrast, Di3a-loaded nanocarriers showed a significant decrease in cell viability when combined with DOX (*p* < 0.01). The highest antiproliferative activity, suggesting synergistic effects, was observed with Di3a-77KL-NC, especially at concentrations of 100 µg/mL and 75 µg/mL, in which cell viability decreased to 27.2% and 61.9%, respectively (CI < 0.9). Di3a-77KL-NLC also shows great results when combined with DOX, with cell viability around 50% at all tested concentrations (CI < 0.9).

A similar trend was observed with treatments using DTX, where free Di3a resulted in nearly 100% cell viability, suggesting an antagonistic effect (CI > 1.1). When in combination with Di3a-77KL-NC, the antitumor drug caused a significant, concentration-dependent decrease in viable cells: 51%, 57.3%, and 64.8%, respectively, for 100, 75, and 50 µg/mL, with CI < 0.9. Additionally, Di3a-77KL-NLC demonstrated dose-dependent effectiveness when combined with DTX, resulting in cell viability of 47.6%, 48.6%, and 51.9%, respectively, along with a CI below 0.9. These findings highlight that encapsulating Di3a enhanced its cytotoxic effectiveness when associated with conventional chemotherapeutic agents after 72 h of incubation (*p* < 0.01). Table 5 shows the tested combinations with DOX or DTX, including CI values and description of the observed effects.

## 4. Discussion

Chemotherapy has a long-standing and recognized role in cancer treatment. Many well-established protocols use combinations of drugs with different mechanisms of action to enhance effectiveness. However, challenges such as systemic toxicity, low selectivity, and acquired resistance often limit the success of antineoplastic therapy [37]. To address these challenges, researchers are actively seeking new, more selective molecules and exploring innovative delivery approaches [27]. Molecules containing selenium have potential as new therapeutic agents in cancer therapy, due to their chemopreventive and antioxidant properties [38].

Therefore, studies are needed to assess whether these molecules present physicochemical properties and ADMET characteristics compatible with satisfactory pharmacological performance. Lipinski’s rules are widely used to estimate oral bioavailability and are not primarily intended for predicting the behavior of intravenously administered drugs. Although Di3a violates some Lipinski parameters, such as molar mass and log P, these violations mainly impact oral absorption and are therefore less critical for intravenous therapies [39,40]. Nevertheless, high molar mass and pronounced lipophilicity remain relevant factors influencing biodistribution, plasma protein binding, metabolic pathways, and potential systemic and immunological toxicity, even for intravenously administered compounds [41]. Highly lipophilic molecules tend to exhibit extensive plasma protein binding and are predominantly metabolized by cytochrome P450 enzymes, which may affect clearance and toxicity profiles. In this context, encapsulation into nanostructured delivery systems represents a rational strategy to modulate these pharmacokinetic limitations, potentially reducing nonspecific distribution and mitigating toxicity while preserving therapeutic efficacy [42].

Nanotechnology has become a promising area of study, aiming to enhance the precision of drug delivery. It also offers numerous benefits, including improved bioavailability and stability, while helping to reduce toxicity, thereby making treatments safer and more effective [43].

We successfully obtained the NCs and NLCs studied using nanoprecipitation and spontaneous emulsification, respectively. Although spontaneous emulsification is not the primary method for preparing NLCs, it provides several benefits: (1) a simple process with reduced equipment expenses, (2) operation at moderate or low temperatures that avoid thermal degradation of the active ingredient, and (3) efficient incorporation of lipophilic compounds [31]. Consequently, it is well-supported in the literature and serves as a practical alternative for the preparation of NLCs [44,45,46]. Particle size and PDI matched with the values commonly reported in the literature for both systems, with the NLCs being significantly larger than the NCs, which reflects the core characteristics of the different systems. Additionally, AFM analysis confirms the size and spherical shape of the nanoformulations. Size around 100–200 nm, facilitates NPs to cross and remain in tumor tissue, known as EPR effect [47,48]. NCs and NLCs showed DL% values consistent with the literature for each matrix type, along with high EE% values [49,50]. Both NPs carry a negative charge compatible with their respective matrices, and the negatively charged surfaces contribute to the formulation, thereby reducing plasma protein adsorption and nonspecific cellular uptake [51]. In general, all formulations showed appropriate physicochemical parameters, with low variation among replicates in the analyzed parameters. This consistency is evidenced by the low SD values, highlighting the suitability of the NP preparation method.

The negative zeta potential in the developed NCs is due to carboxyl groups from PLGA on their surface [52]. Furthermore, in NLCs, compritol’s residual carboxyl groups or traces of free fatty acids tend to ionize, releasing protons and leaving the surface negatively charged [53]. The low modulus value can be attributed to the non-ionic surfactants used in the formulations, such as Pluronic^®^ F-127, Span^®^ 80, and Poly(vinyl alcohol)^®^) [54]. Pluronic F-127 also decreases recognition by complement system proteins in the spleen and liver, and inhibits P-gp by depleting intracellular ATP and reducing mitochondrial transmembrane potential. Thus, it can help to modulate multiple resistance mechanisms, thereby enhancing intracellular drug accumulation and improving therapeutic outcomes [55].

The validated RP-LC method enabled the accurate determination of the EE% and total Di3a content in both NP suspensions. Formulations with high EE% offer several benefits, including the controlled release of the active ingredient for sustained release kinetics, as well as preventing premature release of the active ingredient. They also have lower systemic toxicity since the active ingredient is less available outside the therapeutic target. Additionally, they increase the stability of the active ingredient by protecting it inside the particle from oxidizing and hydrolytic agents [56].

The effect of pH on NP-triggered disruption of the membrane lipid bilayer was assessed using human erythrocytes as a model for endosomal membranes. The results of the pH-dependent hemolysis assay suggest that the incorporation of 77KL into the NPs’ matrix may be linked to a response to pH changes. This effect was more pronounced at pH 5.4 and remains consistent after Di3a was encapsulated in both types of nanosystems, supporting previous findings that NPs with 77 KL or 77 KS may exhibit increased membrane-lytic activity at pH 5.4 [57,58]. At pH 5.4, the increase in hemolytic activity is probably due to a shift in the hydrophobic/hydrophilic balance of 77KL. This shift makes it more likely to interact with the cell membrane. Another possible explanation is that the increased pKa value of the lysine carboxyl group causes a change in protonation state, which may improve membrane binding and subsequently raise hemolytic activity [59]. Additionally, in the case of NLCs, one possible explanation for the lower hemolytic activity at pH 5.4 is the higher concentration of oil present in the core of the system, which may interact with the surfactant at the interface through its fatty acid regions. When these regions have similar aliphatic chains and degrees of unsaturation, there is greater interaction between molecules, making it harder for the 77KL surfactant to detach from the interface and interact with the red blood cell membrane [60].

A clear understanding of protein corona formation is fundamental to predicting the behavior of NPs in biological environments, encompassing aspects of safety and efficacy [61]. In this study, only Di3a-77KL-NC in DMEM with 5% FBS increased in size after 72 h, indicating the NPs’ binding to medium culture proteins. This is expected and happens rapidly when NPs enter a biological system; however, significant increases are undesirable because they may trigger inflammatory and immunogenic responses [62]. Conversely, Di3a-77KL-NC in plasma, and Di3a-77KL-NLC in both water and plasma, showed a significant difference after 72 h, with all displaying a decrease in particle size. Studies assessing liposome size via the protein corona assay state that membrane-impermeable proteins on the NPs generate osmotic pressure, which draws water out of the NPs core and causes the vesicle to shrink, thereby supporting previous findings [63].

To evaluate the biological safety of non-encapsulated and encapsulated Di3a across different nanosystems, in vitro strategies were combined, including the use of non-tumor cells and isolated human blood erythrocytes, as multiple techniques help ensure that the results are reliable and precise [64]. All samples were non-hemolytic at the tested concentrations, as also observed in similar studies [65], indicating the hemocompatibility of the formulations. Supporting the hemolysis assay results, even after adding Di3a into the formulations, the nanocarriers were not toxic to the non-tumor cells. In fact, at higher concentrations, they promoted cell proliferation, indicating their biocompatibility and selective cytotoxicity toward tumor cells.

Additionally, no significant difference in cytotoxicity was found between the bioactive-loaded and the empty NPs. The biocompatibility of PLGA NPs with fibroblast cells has also been reported in other studies, with empty nanocarriers exhibiting cell viability greater than 80% [66]. Furthermore, Compritol solid lipid NPs did not exhibit cytotoxicity in 3T3 cells after 24 h of incubation, regardless of the amount of lipid matrix. However, after encapsulating resveratrol, a slight increase in toxicity was noted [67]. Compared with data previously reported in the literature, the NPs produced in this study showed more promising results, as the formulations were non-cytotoxic to healthy cells after incorporation of the active ingredient, and even promoted cell proliferation.

The MTT assay is a standard method for evaluating cell viability and antiproliferative effects in screenings of new molecules, appreciated for its simplicity, reproducibility, and low cost compared to other techniques [68]. This study applied the MTT assay to evaluate the biocompatibility against normal cells, as well as the cytotoxicity of formulations against cancer cell lines. The evaluations included the effects of NPs as single treatments and in combination with conventional antitumor drugs, to explore potential synergistic interactions.

Both NPs containing Di3a demonstrated higher toxicity compared to non-encapsulated Di3a, with a dose-dependent effect observed in A549 cells after 72 h. In the resistant/MDR cell line, there was a less remarkable reduction in cell viability at the same concentrations. Nevertheless, the effect was even more pronounced when compared to the non-encapsulated compound. These data highlight the intrinsic resistance of the cell line.

As the formulations as single treatments already exhibited significant cytotoxicity in A549 cells, the synergistic effect with DOX or DTX was not assessed in this cell line. Instead, this effect was investigated in the NCI/ADR-RES cell line, aiming to sensitize these MDR cells. These cells were mistakenly thought to come from breast cancer for nearly twenty years, but genotypic analysis at NCI confirmed they are derived from human ovarian carcinoma cells (OVCAR-8) [69,70]. It has a stable, strong MDR phenotype with cross-resistance to many chemotherapeutic agents, mainly due to overexpression and activity of P-gp (ABCB1/MDR1), which increases drug efflux. Because of these traits, NCI/ADR-RES is widely used in the NCI-60 drug screening and MDR research, making it a key cancer model [71].

The NCI/ADR-RES cell line is intrinsically resistant to DOX and taxanes due to the overexpression of efflux pumps, such as P-gp [72]. The in vitro findings supported the in silico data, suggesting that Di3a is probably a P-gp substrate, but not a P-gp inhibitor. Consequently, it did not expressively sensitize this cell line, even when encapsulated, owing to the cells’ inherent resistance mechanism. Therefore, this study explored different approaches of combining nanocarriers loaded with an innovative organoselenium and conventional antitumor drugs to overcome this resistance. Fixed concentrations of DOX and DTX were employed as an initial screening strategy to assess the interactions between the NPs and these drugs. Subtoxic concentrations were chosen to support a translational approach aimed at lowering doses of highly toxic chemotherapeutics and reducing systemic side effects. Additionally, using subtoxic doses prevents the high cytotoxicity of one drug from masking the interaction effects.

The CI, based on the Chou–Talalay method, measures drug interactions by directly comparing the observed combination effect with the expected additive response. Since this approach includes dose–response relationships and potency measures, it reduces subjective judgments that rely only on cell viability percentages. Consequently, CI values provide more reliable evidence of the type of drug combination effect [35,36]. Non-encapsulated Di3a, in combination with DOX/DTX, suggested strong or very strong antagonistic effects across all tested conditions, indicating that, in its non-encapsulated form and even when combined with other drugs, it is not able to sensitize resistant cells. On the other hand, nanoencapsulation of Di3a changed the observed effects, and markedly synergistic antitumor effects were suggested. The synergism between NPs and DOX was stronger than with DTX, leading to a marked decrease in cell viability. It is noteworthy that several studies have demonstrated that endocytosis represents the primary pathway for cellular uptake of NPs. Being an active and quick process, this internalization likely leads to a higher intracellular concentration of the encapsulated drug compared to the unloaded solution [73,74].

The compound Di3a was inspired by thymidine, a nucleoside analog designed to resemble natural nucleosides but with specific molecular modifications that disrupt DNA replication in cells. To activate it, the nucleoside must be phosphorylated into a nucleotide, which then can inhibit DNA strand elongation by acting during the DNA synthesis of the cell cycle [75]. DOX, an anthracycline drug, exerts its antitumor effects through multiple mechanisms that induce DNA damage and apoptosis. These include (1) intercalating into DNA to disrupt replication and transcription; (2) inhibiting topoisomerase II, resulting in double-strand DNA breaks; and (3) causing oxidative damage to cellular DNA, resulting in cell cycle arrest at the S and G2 phases [76]. Regarding DTX, a taxane and antimitotic agent, its mechanism of action includes promoting tubulin polymerization and preventing microtubule depolymerization. This interferes with the cell’s ability to complete mitosis, affecting the G2/M phase of the cell cycle and leading to apoptosis [77].

The interaction between these classes of drugs can produce varying effects, from antagonism to synergy. These responses are influenced by how their mechanisms of action affect cellular metabolism and DNA integrity. Combining nucleoside analogs with anthracyclines can increase cytotoxicity if the depletion of intracellular deoxynucleotides and the resulting replicative stress from the analog enhance the oxidative and genotoxic damage caused by the anthracycline. However, if DNA repair pathways or cell cycle checkpoints are activated, they may reduce nucleoside analog incorporation into DNA and weaken the overall cytotoxic effect, resulting in a less synergistic or even antagonistic interaction [78]. More precisely, if DOX acts first and activates DNA repair pathways or cell cycle checkpoints, it may decrease nucleoside incorporation into DNA and reduce cytotoxicity, leading to antagonism. Similarly, nucleoside analogs hinder DNA synthesis and replication, while taxanes interfere with microtubule dynamics and mitosis. The overall response, whether synergistic or antagonistic, is likely influenced by the interaction between these pathways and their impact on cell cycle control, DNA stability, and the balance between repair and apoptosis. Effectively, the presence of DTX before Di3a might induce a G2/M arrest, which could block cells from entering the S phase in which Di3a acts. This may decrease the overall effectiveness of the combination and could justify the antagonism with non-encapsulated Di3a. Therefore, the effects of these agents may differ depending on how closely their mechanisms align [79]. Taken together, these findings suggest that the synergistic effects observed for Di3a-loaded nanocarriers combined with DOX or DTX may result from the sustained intracellular release provided by nanoencapsulation, which likely enhances the coordination of mechanisms that induce tumor cell death. In this sense, it seems that encapsulating Di3a enhances its uptake by cells through endocytosis, extending its effectiveness and stabilizing its metabolism. Additionally, it synchronizes the cell cycle with the timing of DOX/DTX action, which helps to sensitize resistant cells.

Similar studies also observed the synergistic effect between different NP-loaded with innovative nucleoside analogs when combined (1) with non-encapsulated DOX [80], and (2) co-encapsulated with paclitaxel [65]. In both studies, the combination was able to sensitize resistant cells, highlighting the importance of combined therapies.

As part of this investigation, we utilized both in silico platforms and in vitro assays to assess the safety and potential antineoplastic properties of Di3a. While in silico approaches are common, their predictive results still require experimental validation. Likewise, drug release under varying pH conditions was not investigated in this study, representing a limitation that warrants further research to confirm the pH-responsive behavior of the NPs. Another key challenge is simulating the tumor microenvironment, which comprises neoplastic cells, stromal cells, connective tissue, and immune cells—a complex heterogeneity that is hard to replicate in monolayer cell assays. Additionally, exploring the molecular mechanisms behind the cytotoxic response of individual and combined therapies (Di3a-NPs vs. Di3a-NPs + DOX/DTX), including cell cycle and apoptosis studies, as well as internalization and intracellular distribution, is crucial. Comprehensive mechanistic validation is beyond the scope of the current manuscript and will be addressed in future publications. Although Di3a encapsulation shows promise as a therapeutic candidate, further research is necessary to corroborate its efficacy and safety.

## 5. Conclusions

In this study, two drug delivery systems were successfully developed using biocompatible and biodegradable matrices. Both NPs demonstrated suitable physicochemical properties and were non-toxic to non-tumor cells, emphasizing their safety and selectivity for target tumor cells. Furthermore, the findings achieved on the membranolytic assays indicate a potential pH-responsive behavior of the NPs, and drug release evaluations under varying pH conditions are necessary to substantiate this premise. The nanoencapsulation of Di3a significantly enhanced its cytotoxic potential in A549 cells compared to non-encapsulated Di3a. The overall limited cytotoxicity observed in NCI/ADR-RES cells reflects the robust drug resistance mechanisms present in this cell line. Nevertheless, the synergism studies with DOX and DTX suggested that nanoencapsulation facilitated the coordination of the mechanisms of action between the compounds, leading to a marked increase in the antiproliferative effects in this cell line, suggesting the sensitization of MDR cells. Finally, despite some limitations in Di3a in silico property prediction, the study highlights the potential of combining organoselenium-loaded NPs with conventional antitumor drugs as a strategy to sensitize MDR in cancer, while also emphasizing the safety of the developed formulations.

## Figures and Tables

**Table 1:** Physicochemical properties of Di3a compared across different platforms.

Parameter	Molinspiration	pKCSM	SwissADME
Hydrogen bond acceptor (HBA)	15 *	13 *	9
Hydrogen bond donor (HBD)	4	4	4
Molar Mass (MM)	886 g/mol *	886 g/mol *	886 g/mol *
Logarithm Octanol/water partition coefficient (LogP)	5.66 *	-	5.24 *
Polar Surface Area (TPSA)	191 Å^2^	-	191 Å^2^
Number of rotatable bonds (Nrotb)	12	12	12

Properties that violated Lipinski’s rules are indicated with *.

**Table 2:** Comparative Pharmacokinetic and Toxicological Properties of Di3a obtained from different data platforms.

Comparative Pharmacokinetic and Toxicological Properties of Di3a				
Category	pKCSM	admetSAR	SwissADME	ProTox
P-glycoprotein	Substrate; P-gp II inhibitor	Non-substrate	Substrate	-
BBB permeability	−1.8 (low)	(0.6)	No	Active (0.6)
Volume of distribution	−0.4 (log L/kg) Low	-	-	-
Plasma protein binding	High binding			
CYP3A4 (substrate)	Yes	Yes	-	-
CYP3A4 (inhibitor)	Yes	Yes	-	-
OCT2 (renal)	No	Non-substrate	-	-
Total Clearance	−0.7 log/mL/min/kg	-	-	-
AMES toxicity	Negative	Negative	-	Inactive
Carcinogenicity	No	No	-	Inactive
Hepatotoxicity	No	-	-	Active (0.5)
Neurotoxicity	-	-	-	Active (0.9)
Nephrotoxicity	-	-	-	Active (0.5)
hERG inhibition	hERG I: No; hERG II: Yes	-	-	-

**Table 3:** Physicochemical characterization of nanoparticles. SD, standard deviation (*n* = 3).

	Particle Size (nm) ± SD	PDI ± SD	ZP (mV) ± SD	pH ± SD	Di3a Content (mg/mL) ± SD	EE (%) ± SD	DL (%) ± SD
77KL-NC	116 ± 2	0.14 ± 0.01	−5.2 ± 0.8	5.7 ± 0.2	-	-	-
Di3a-77KL-NC	120 ± 3	0.08 ± 0.01	−6.0 ± 2.1	6.1 ± 0.3	3.06 ± 0.07	99.9 ± 0.1	7.2 ± 0.2
77KL-NLC	217 ± 3	0.11 ± 0.02	−5.6 ± 1.8	6.2 ± 0.2	-	-	-
Di3a-77KL-NLC	259 ± 11	0.10 ± 0.04	−5.0 ± 0.8	6.6 ± 0.2	2.50 ± 0.09	99.9 ± 0.1	4.0 ± 0.1

**Table 4:** Cytotoxicity expressed as IC_50_ in A549 and NCI/ADR-RES cell lines.

IC_50_ Values (µg/mL)		
	Cell Lines	
	A549	NCI/ADR-RES
Non-encapsulated Di3a	>100	>100
Di3a-77KL-NC	81.08	>100
Di3a-77KL-NLC	30.14	>100
77KL-NC	>100	>100
77KL-NLC	>100	>100

**Table 5:** CI and description of the observed effects when combined with different antitumor agents.

	CI	Description
Non-encapsulated Di3a 100 µg/mL + DOX	5.43	Strong antagonism
Non-encapsulated Di3a 75 µg/mL + DOX	>10	Very strong antagonism
Non-encapsulated Di3a 50 µg/mL + DOX	>10	Very strong antagonism
Di3a-77KL-NC 100 µg/mL + DOX	0.009	Very strong synergism
Di3a-77KL-NC 75 µg/mL + DOX	0.333	Synergism
Di3a-77KL-NC 50 µg/mL + DOX	1.88	Slight antagonism
Di3a-77KL-NLC 100 µg/mL + DOX	0.325	Synergism
Di3a-77KL-NLC 75 µg/mL + DOX	0.240	Strong synergism
Di3a-77KL-NLC 50 µg/mL + DOX	0.184	Strong synergism
Non-encapsulated Di3a 100 µg/mL + DTX	>10	Very strong antagonism
Non-encapsulated Di3a 75 µg/mL + DTX	>10	Very strong antagonism
Non-encapsulated Di3a 50 µg/mL + DTX	>10	Very strong antagonism
Di3a-77KL-NC 100 µg/mL + DTX	0.206	Strong synergism
Di3a-77KL-NC 75 µg/mL + DTX	0.361	Synergism
Di3a-77KL-NC 50 µg/mL + DTX	0.493	Synergism
Di3a-77KL-NLC 100 µg/mL + DTX	0.413	Synergism
Di3a-77KL-NLC 75 µg/mL + DTX	0.372	Synergism
Di3a-77KL-NLC 50 µg/mL + DTX	0.516	Synergism

## Data Availability

The original contributions presented in this study are included in the article and Supplementary Material. Further inquiries can be directed to the corresponding author.

## Supplementary Materials

The supporting information regarding physicochemical characterization and stability of NPs, and the validation of the HPLC method can be downloaded from: https://www.mdpi.com/article/10.3390/pharmaceutics18010057/s1. Table S1. Individual values for each replicate in the physicochemical characterization of NPs. Table S2. Physicochemical stability parameters of NPs evaluated at initial time point, and 7, 14 and 30 days after preparation. Figure S1. Assessment of the method’s robustness. A (injection volume), B (flow), C (acetonitrile). Parameters assessed using a three-factor, two-level factorial design did not significantly affect the test results (*p* > 0.05), as indicated by the Pareto Chart of the Standardized Effects. Figure S2: (A) PDA absorption spectra of Di3a obtained by the HPLC method and (B) Di3a peak with a purity index exceeding 0.999, showing no interference during analysis. Figure S3: Chromatograms recorded during the selectivity assessment of the HPLC method for Di3a in the analyzed nanoformulation. (A) Dia-77KL-NC and 77-KL-NC, and (B) Dia-77KL-NLC and 77KL-NLC, respectively. The absence of interfering peaks at the analyte retention times confirms the method’s selectivity. (C) Di3a standard solution. Table S3. Accuracy of the analytical method, expressed as the percentage recovery at different concentration levels (80–120%). Table S4. The precision confirmed by relative standard deviation values below the 2% acceptance limit for repeatability, inter-day assessments, and between analysts assessments. Figure S4. Linearity in the concentration range of 10 to 60 µg/mL, with significant linear regression (Fcalc = 14,878.76 > Fcritical = 4.75, *p* < 0.05) and no deviation from linearity (Fcalc = 0.41 < Fcritical = 3.26, *p* > 0.05).

## Abbreviations

The following abbreviations are used in this manuscript:

77KL	N^α^, N^ε^-dioctanoyl lysine with an inorganic lithium counterion
A549	Human lung adenocarcinoma
AFM	Atomic force microscopy
BCRJ	Rio de Janeiro cell bank
CI	Combination index
Di3a	5′-Seleno-(phenyl)-(3-N)-5′-Seleno-(4-methylphenyl)-4-N(6-chloro-1,3,5-triazin-2-yl)-thymidine
DMEM	Dulbecco’s Modified Eagle’s Medium
DMSO	Dimethyl sulfoxide
DOX	Doxorubicin
DTX	Docetaxel
EPR	Enhanced permeability and retention effect
FBS	Fetal bovine serum
HPLC	High-performance liquid chromatography
L929	Murine fibroblast cell line
MCT	Medium-chain triglycerides
MDR	Multidrug resistance
MTT	2,5-diphenyl-3-(4,5-dimethyl- 2-thiazolyl) tetrazolium bromide
NCI/ADR-RES	Multidrug-resistant human ovarian carcinoma
NCs	PLGA nanocapsules
NLCs	Nanostructured lipid carriers
NPs	Nanoparticles
PBS	Phosphate-buffered saline
PDI	Polydispersity index
PLGA	Poly(lactic-co-glycolic acid)
SD	Standard deviation
SE	Standard error
ZP	Zeta potential
