# Supplementary material for: Preclinical Investigation of PLGA Nanocapsules and Nanostructured Lipid Carriers for Organoselenium Delivery: Comparative In Vitro Toxicological Profile and Anticancer Insights
Source: Pharmaceutics. 2025 Dec 31;18(1):57. doi: 10.3390/pharmaceutics18010057 (PMC12844722; doi:10.3390/pharmaceutics18010057)
Supplement: Supplementary file 1 [file pharmaceutics-18-00057-s001.zip › pharmaceutics-4017079-supplementary.pdf]

pharmaceutics

MDPI

*Supplementary Materials*

# Preclinical Investigation of PLGA Nanocapsules and Nanostructured Lipid Carriers for Organoselenium Delivery: Comparative In Vitro Toxicological Profile and Anticancer Insights

**Bianca Costa Maia-do-Amaral [1], Taís Baldissera Pieta [1], Luisa Fantoni Zanon [1], Gabriele Cogo Carneosso [1], Laísa Pes Nascimento [1], Nayra Salazar Rocha [2], Bruna Fracari do Nascimento [1], Letícia Bueno Macedo [3], Tielle Moraes de Almeida [4], Oscar Endrigo Dorneles Rodrigues [2], Scheila Rezende Schaffazick [5], Clarice Madalena Bueno Rolim [1] and Daniele Rubert Nogueira-Librelotto [1,*]**

[1] Laboratório de Testes e Ensaios Farmacêuticos In Vitro, Departamento de Farmácia Industrial, Universidade Federal de Santa Maria, Av. Roraima 1000, Santa Maria 97105-900, Brazil; bianca.maia@acad.ufsm.br (B.C.M.-d.-A.); tais.pieta@acad.ufsm.br (T.B.P.); luisa.zanon@acad.ufsm.br (L.F.Z.); gabriele.cogo@acad.ufsm.br (G.C.C.); laisa.pes@acad.ufsm.br (L.P.N.); bruna.fracari@acad.ufsm.br (B.F.d.N.); clarice.rolim@ufsm.br (C.M.B.R.)
[2] Departamento de Química, Universidade Federal de Santa Maria, Av. Roraima 1000, Santa Maria 97105-900, Brazil; nayrarocha0012@gmail.com (N.S.R.); rodriguesoed@gmail.com (O.E.D.R.)
[3] Laboratório de Engenharia e Processos Químicos, Universidade Federal de Santa Maria, Av. Roraima 1000, Santa Maria 97105-900, Brazil; leticiabuenomacedo@gmail.com
[4] Departamento de Físico-Química, Universidade Federal de Santa Maria, Av. Roraima 1000, Santa Maria 97105-900, Brazil; tielle.almeida@ufsm.br
[5] Departamento de Farmácia Industrial, Universidade Federal de Santa Maria, Av. Roraima 1000, Santa Maria 97105-900, Brazil; scheila.schaffazick@ufsm.br
* Correspondence: librelotto.daniele@ufsm.br

**Table S1.** Individual values for each replicate in the physicochemical characterization of NPs.

| | Particle Size (nm) | PDI | ZP (mV) | pH | Di3a Content (mgmL) | EE (%) | DL (%) |
|---|---|---|---|---|---|---|---|
| **77KL-NC** | 116 | 0.14 | -5.4 | 5.9 | | | |
| | 117 | 0.13 | -4.4 | 5.7 | - | - | - |
| | 114 | 0.15 | -5.9 | 5.6 | | | |
| Mean ± SD | 116 ± 2 | 0.14 ± 0.01 | -5.2 ± 0.8 | 5.7 ± 0.2 | - | - | - |
| **Di3a-77KL-NC** | 117 | 0.07 | -4.7 | 6.0 | 3.13 | 99.82 | 7.4 |
| | 119 | 0.09 | -8.4 | 5.9 | 3.05 | 99.98 | 7.3 |
| | 123 | 0.09 | -5.0 | 6.10 | 2.95 | 99.90 | 7.0 |
| Mean ± SD | 120 ± 3 | 0.08 ± 0.01 | -6.0 ± 2.1 | 6.0 ± 0.1 | 3.05 ± 0.09 | 99.9 ± 0.1 | 7.2 ± 0.2 |
| **77KL-NLC** | 214.8 | 0.10 | -5.60 | 6.2 | | | |
| | 217.3 | 0.12 | -6.9 | 6.4 | - | - | |
| | 219.8 | 0.11 | -4.30 | 6.0 | | | |
| Mean ± SD | 217.3 ± 3 | 0.11 ± 0.01 | -5.60 ± 1.3 | 6.2 ± 0.2 | - | - | |
| **Di3a-77KL-NLC** | 246 | 0.089 | -4.8 | 6.7 | 2.59 | 99.99 | 4.1 |
| | 264 | 0.089 | -4.3 | 6.4 | 2.42 | 99.92 | 3.9 |
| | 267 | 0.150 | -5.9 | 6.6 | 2.46 | 99.79 | 3.9 |
| Mean ± SD | 259 ± 11 | 0.109 ± 0.04 | -5.0 ± 0.8 | 6.6 ± 0.2 | 2.5 ± 0.09 | 99.9 ± 0.1 | 4.0 ± 0.1 |

**Table S2.** Physicochemical stability parameters of NPs evaluated at initial time point, and 7, 14 and 30 days after preparation.

| | Time | Particle Size (nm) ± SD | PDI ± SD | ZP (mV) ± SD | pH ± SD | Di3a Content (mg/mL) ± SD |
|---|---|---|---|---|---|---|
| **77KL-NC** | 0 | 116 ± 2 | 0.14 ± 0.01 | -5.2 ± 0.8 | 5.7 ± 0.2 | - |
| | 7 | 118 ± 1 | 0.14 ± 0.03 | -6.3 ± 1.4 | 5.7 ± 0.2 | - |
| | 14 | 121 ± 1 | 0.14 ± 0.01 | -6.1 ± 1.2 | 5.5 ± 0.03 | - |
| | 30 | 122 ± 7 | 0.14 ± 0.03 | -2.7 ± 0.3 | 4.9 ± 0.01 | - |
| **Di3a-77KL-NC** | 0 | 120 ± 3 | 0.08 ± 0.01 | -6.0 ± 2.1 | 6.1 ± 0.3 | 3.06 ± 0.07 |
| | 7 | 122 ± 7 | 0.11 ± 0.03 | -5.31 ± 2 | 5.9 ± 0.4 | 2.97 ± 0.05 |
| | 14 | 121 ± 7 | 0.10 ± 0.02 | -5.4 ± 1.7 | 5.3 ± 0.3 | 2.92 ± 0.08 |
| | 30 | 124 ± 5 | 0.12 ± 0.03 | -7.9 ± 1 | 5.5 ± 0.3 | 2.84 ± 0.08 |
| **77KL-NLC** | 0 | 217 ± 3 | 0.11 ± 0.02 | -5.6 ± 1.8 | 6.2 ± 0.2 | - |
| | 7 | 230 ± 4 | 0.18 ± 0.08 | -5.8 ± 0.5 | 6.4 ± 0.2 | - |
| | 14 | 226 ± 4 | 0.14 ± 0.05 | -4.8 ± 0.2 | 6.4 ± 0.1 | - |
| | 30 | 266 ± 9 | 0.22 ± 0.01 | -5.9 ± 0.7 | -5.9 ± 0.1 | - |
| **Di3a-77KL-NLC** | 0 | 259 ± 11 | 0.109 ± 0.04 | -5.0 ± 0.8 | 6.6 ± 0.2 | 2.50 ± 0.09 |
| | 7 | 248 ± 11 | 0.16 ± 0.05 | -4.2 ± 1.4 | 6.9 ± 0.3 | 2.36 ± 0.2 |
| | 14 | 247 ± 4 | 0.19 ± 0.06 | -4.6 ± 1.1 | 7.1 ± 0.3 | 2.24 ± 0.08 |
| | 30 | 265 ± 1 | 0.17 ± 0.04 | -7.2 ± 2.1 | 7.2 ± 0.7 | 2.37 ± 0.02 |

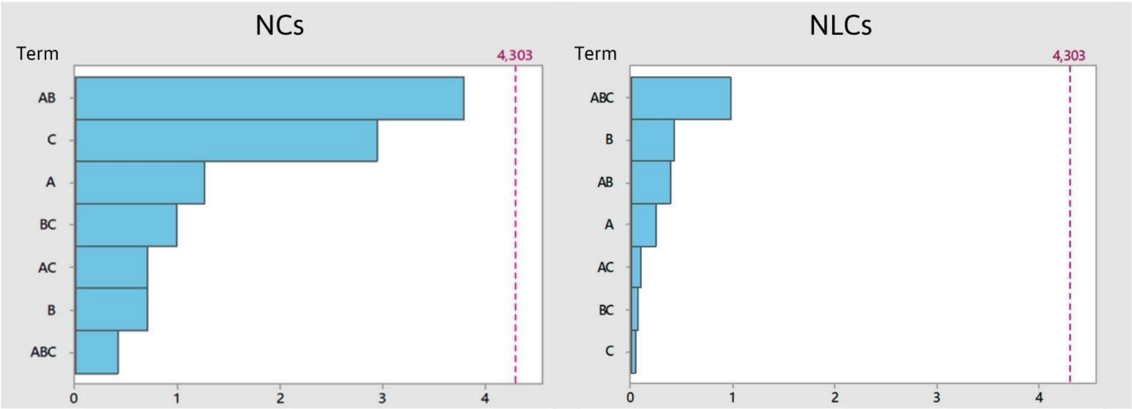


**Figure S1. Assessment of the method's robustness.** A (injection volume), B (flow), C (acetonitrile). Parameters assessed using a three-factor, two-level factorial design did not significantly affect the test results ($p > 0.05$), as indicated by the Pareto Chart of the Standardized Effects.

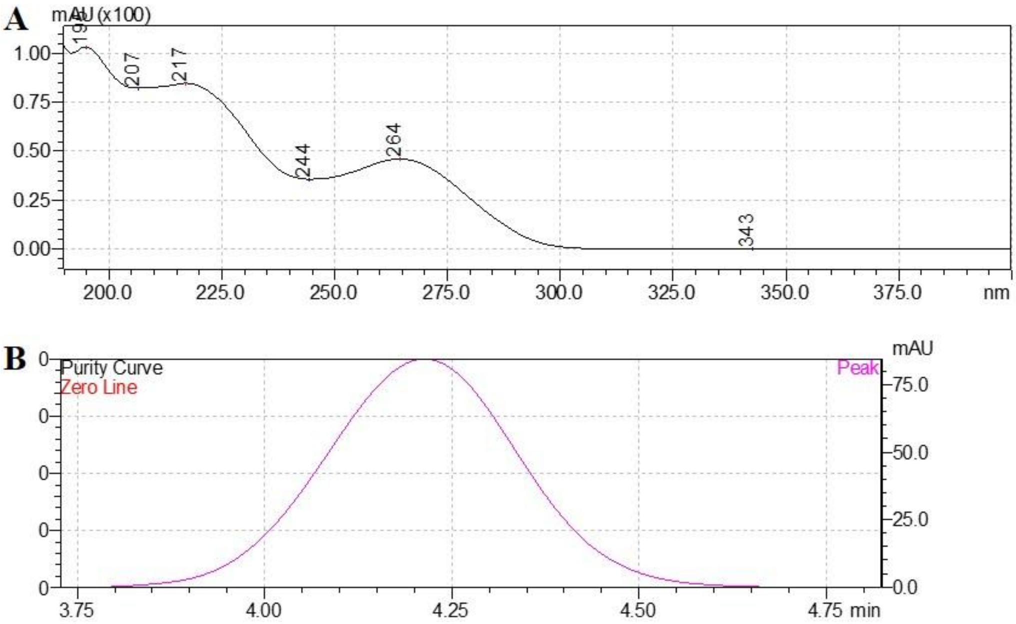


**Figure S2:** (A) PDA absorption spectra of Di3a obtained by the HPLC method and (B) Di3a peak with a purity index exceeding 0.999, showing no interference during analysis.

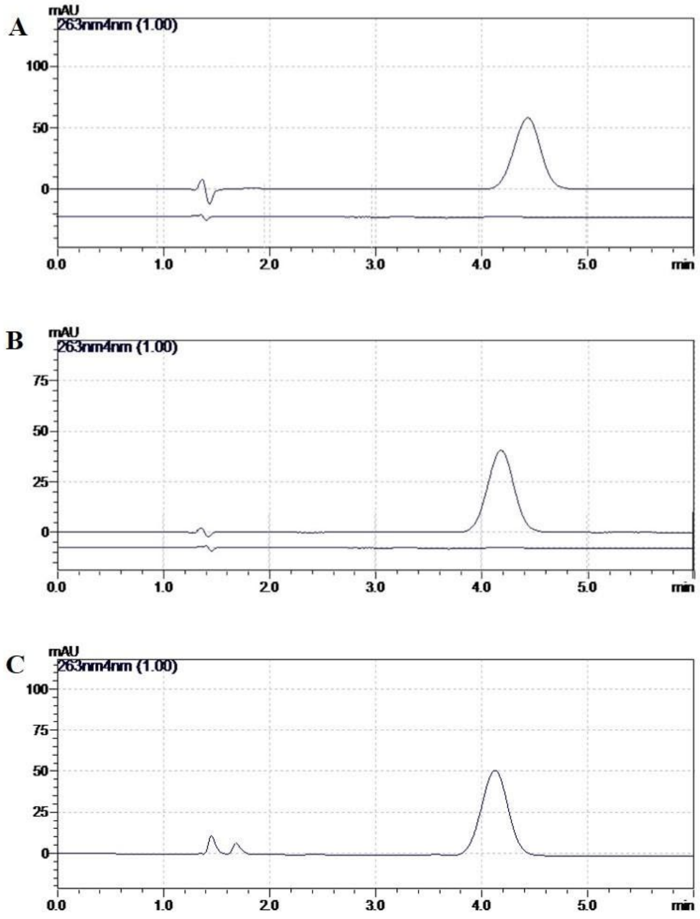


**Figure S3:** Chromatograms recorded during the selectivity assessment of the HPLC method for Di3a in the analyzed nanoformulation. (A) Dia-77KL-NC and 77-KL-NC, and (B) Dia-77KL-NLC and 77KL-NLC, respectively. The absence of interfering peaks at the analyte retention times confirms the method's selectivity. (C) Di3a standard solution.

**Table S3.** Accuracy of the analytical method, expressed as the percentage recovery at different concentration levels (80–120%).

| | Accuracy | | | |
|---|---|---|---|---|
| | **NCs** | **Mean Recovery** | **NLCs** | **Mean Recovery** |
| **R1** | 101,27% | 100,81% | 98,31% | 98,61% |
| | 100,36% | | 98,91% | |
| **R2** | 100,90% | 100,76% | 98,41% | 99,34% |
| | 100,62% | | 100,26% | |
| **R3** | 101,22% | 101,46% | 98,52% | 98,46% |
| | 101,70% | | 98,39% | |
| **Mean** | | 101,01% | | 98,80% |

**Table S4.** The precision confirmed by relative standard deviation (RSD) values below the 2% acceptance limit for repeatability, inter-day, and between analysts assessments.

| | Precision | |
|---|---|---|
| | **Relative Standard Deviation (RSD)** | |
| | **NCs** | **NLCs** |
| **Repeatability** | 1.29 | 0.92 |
| **Interday** | 0.97 | 0.84 |
| **Between-analyst** | 0.92 | 1.3 |

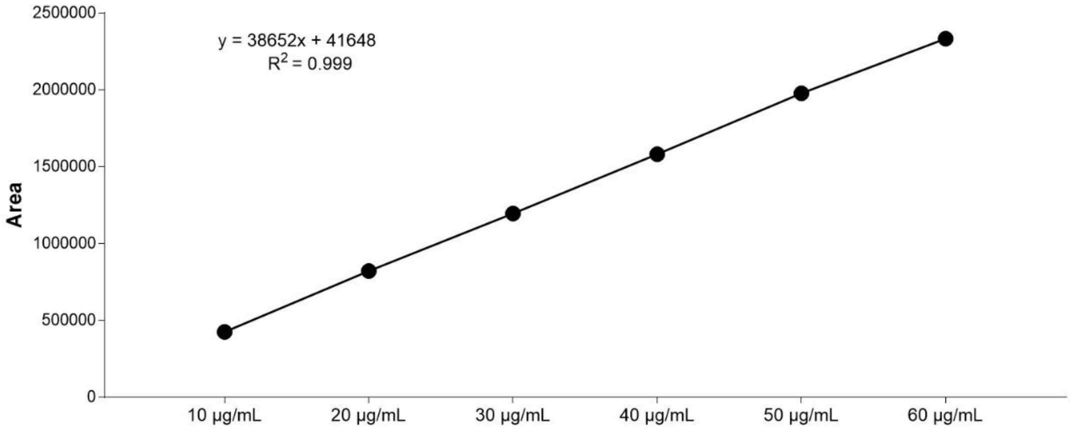


**Figure S4.** Linearity in the concentration range of 10 to 60 µg/mL, with significant linear regression ($F_{calc}$ = 14,878.76 > $F_{critical}$ = 4.75, $p < 0.05$) and no deviation from linearity ($F_{calc}$ = 0.41 < $F_{critical}$ = 3.26, $p > 0.05$).
